# Non-specific mechanisms in orthodox and CAM management of low back pain (MOCAM): theoretical framework and protocol for a prospective cohort study

**DOI:** 10.1136/bmjopen-2016-012209

**Published:** 2016-05-27

**Authors:** Katherine Bradbury, Miznah Al-Abbadey, Dawn Carnes, Borislav D Dimitrov, Susan Eardley, Carol Fawkes, Jo Foster, Maddy Greville-Harris, J Matthew Harvey, Janine Leach, George Lewith, Hugh MacPherson, Lisa Roberts, Laura Parry, Lucy Yardley, Felicity L Bishop

**Affiliations:** 1 Department of Psychology, University of Southampton, Southampton, UK; 2 Blizard Institute, Queen Mary University of London, London, UK; 3 Primary Care and Population Sciences, University of Southampton, Southampton, UK; 4 Clinical Research Centre for Health Professions, University of Brighton, Brighton, UK; 5 Health Sciences, University of York, New York, UK; 6 Health Sciences, University of Southampton, Southampton, UK

**Keywords:** COMPLEMENTARY MEDICINE, PAIN MANAGEMENT, PRIMARY CARE, RHEUMATOLOGY

## Abstract

**Introduction:**

Components other than the active ingredients of treatment can have substantial effects on pain and disability. Such ‘non-specific’ components include: the therapeutic relationship, the healthcare environment, incidental treatment characteristics, patients’ beliefs and practitioners’ beliefs. This study aims to: identify the most powerful non-specific treatment components for low back pain (LBP), compare their effects on patient outcomes across orthodox (physiotherapy) and complementary (osteopathy, acupuncture) therapies, test which theoretically derived mechanistic pathways explain the effects of non-specific components and identify similarities and differences between the therapies on patient–practitioner interactions.

**Methods and analysis:**

This research comprises a prospective questionnaire-based cohort study with a nested mixed-methods study. A minimum of 144 practitioners will be recruited from public and private sector settings (48 physiotherapists, 48 osteopaths and 48 acupuncturists). Practitioners are asked to recruit 10–30 patients each, by handing out invitation packs to adult patients presenting with a new episode of LBP. The planned multilevel analysis requires a final sample size of 690 patients to detect correlations between predictors, hypothesised mediators and the primary outcome (self-reported back-related disability on the Roland-Morris Disability Questionnaire). Practitioners and patients complete questionnaires measuring non-specific treatment components, mediators and outcomes at: baseline (time 1: after the first consultation for a new episode of LBP), during treatment (time 2: 2 weeks post-baseline) and short-term outcome (time 3: 3 months post-baseline). A randomly selected subsample of participants in the questionnaire study will be invited to take part in a nested mixed-methods study of patient–practitioner interactions. In the nested study, 63 consultations (21/therapy) will be audio-recorded and analysed quantitatively and qualitatively, to identify communication practices associated with patient outcomes.

**Ethics and dissemination:**

The protocol is approved by the host institution's ethics committee and the NHS Health Research Authority Research Ethics Committee. Results will be disseminated via peer-reviewed journal articles, conferences and a stakeholder workshop.

**Strengths and limitations of this study**

- This study compares multiple non-specific components of treatment to identify those most strongly associated with patient health outcomes.
- Including multiple therapies enables new comparisons of non-specific components of treatment across different therapies.
- The nested mixed-methods study will generate new insight into patient–practitioner communication in low back pain and how it differs by therapeutic modality.
- Practitioners and patients are recruited from diverse settings across the UK, enhancing generalisability of the findings.
- The observational design not only means that non-specific treatment components are being studied as they occur in everyday clinical practice (high ecological/external validity) but also means that causal relationships between variables will not be demonstrated.

## Introduction

Components other than the active ingredients of treatment can have substantial effects on pain and disability. For example, placebo controls in osteoarthritis trials produce a moderate effect size (0.5) on pain compared to a small effect size (0.03) of no-treatment controls.^1^ Such components have been termed ‘non-specific’^2^ and comprise the broad constellation of psychological, social and environmental factors that act alongside and can interact with the ‘specific’ ingredients of treatment. This paper presents a protocol for a mixed-methods cohort study to investigate and compare non-specific components in physiotherapy, osteopathy and acupuncture for patients with low back pain (LBP). The purpose is to identify the most powerful non-specific components in a naturalistic setting and provide a deeper understanding of the pathways through which non-specific components generate positive patient outcomes. This is an essential prerequisite for designing interventions to augment non-specific components and thus enable existing therapies to deliver maximal patient benefit.

Five domains of non-specific components have been proposed: patient–practitioner interaction and relationship, healthcare environment, incidental characteristics of treatment, patients’ beliefs and practitioners’ beliefs.^3^ Evidence suggests that components from each of these domains might mediate or enhance patient outcomes in musculoskeletal and other conditions, as follows. Positive and empathetic patient–practitioner relationships and a strong patient–practitioner alliance are associated with improved patient outcomes.^3–6^ Different healthcare settings foster different patient and practitioner behaviours^7–10^ and influence the magnitude of placebo effects.^11^ ^12^ For example, in hospitals, good organisational environments (eg, collegiate working relationships) enhance patient satisfaction^13^ and the physical–sensory environment (eg, music) can reduce patient anxiety, but evidence is limited and more studies are needed in other settings.^14^ ^15^ Incidental characteristics of treatment such as the number and/or size of pills, colour and cost of medications influence outcomes, but equivalent characteristics in non-pharmacological interventions are not well understood.^16–18^ Patients can experience better treatment outcomes when they have higher expectations of their treatment, believe it to be more credible and adhere to instructions to take medications, therapies, exercises or indeed placebos.^19–23^ Musculoskeletal practitioners differ in their beliefs about pain^24^ and these beliefs affect clinical practice^25^ although individual differences in effect between practitioners have not been well modelled and are difficult to investigate.^26–28^

Complementary and alternative medicine (CAM) practices and practitioners might be particularly skilled at augmenting the context of treatment and thus enhancing patient outcomes.^29^ In particular, patient–practitioner interactions and relationships may be particularly well developed and effective in CAM. For example, the consultation process (alone, but in the context of a placebo or sham treatment) is effective in acupuncture for irritable bowel syndrome^30^ and homeopathy for rheumatoid arthritis.^31^ CAM therapies might also enhance patient outcomes through other so-called non-specific domains. The ritualistic performances involved in different CAM treatments, such as the paraphernalia around needling in acupuncture, may also contribute to their effects.^32^ Patients’ and practitioners’ belief in their chosen CAM therapy and their broader motivations also appear important.^24^ ^33–36^ For example, the effects of acupuncture are partially mediated by psychological factors implicated in the maintenance of musculoskeletal pain, including self-efficacy for coping and fear avoidance beliefs.^37^

Some aspects of patient–practitioner interactions are integral and specific to particular CAM therapies as they operate via theoretically defined, therapy-specific mechanisms: Examples from acupuncture include traditional diagnostic techniques, talk about traditional acupuncture models for understanding pain and the associated, theoretically driven, lifestyle advice that acupuncturists offer.^38–42^ Qualitative studies illustrate how homeopaths and acupuncturists communicate empathetically within consultations to empower and support patients to cope with illness and thus encourage positive, theoretically driven, lifestyle changes.^43–45^ Thus non-specific components of CAM (such as supportive patient–practitioner relationships) may influence patient outcomes at least in part by augmenting the specific effects of theoretically driven treatment components.

The healthcare environment probably contributes to the strong non-specific components attributed to CAM. Much CAM research has been conducted in private sector (or clinical trial) settings, which may be inherently better able to augment non-specific treatment components than public sector settings. For example, qualitative evidence suggests that private settings facilitate clinical autonomy, support longer consultation times and shorter waiting lists, engender more consumerist and/or collaborative relationships, and provide more attractive and convenient physical environments compared to public sector settings.^8^ ^9^ However, healthcare sector might not have a uniform influence on clinical practice across therapies. For example, osteopaths (but not physiotherapists) working in the National Health Service (NHS) retained some positive non-specific components more characteristic of the private sector, such as mutualistic and supportive therapeutic relationships and longer consultation times.^10^ Thus NHS environments might create larger differences between CAM and orthodox therapies than do exist in private sector environments. While the UK-BEAM study found no effect of the healthcare sector on exercise and manipulation outcomes,^46^ the clinical trial setting might have dominated the organisational context and concealed any effects of sector.^7^

Overall the evidence suggests that non-specific components can enhance patient outcomes and CAMs may be potent at optimising non-specific components. However, most studies have focused on one or two components, meaning that we do not understand their relative importance or how they interact with each other. Most studies have focused on a single therapy, which makes it difficult to be confident that the effects are indeed due to non-specific components shared across therapies rather than being intimately entwined with a particular therapy's theoretical framework.^38^ There is also a need for more theoretical work explaining how non-specific components elicit positive effects.^47^ ^48^ This study therefore aims to examine non-specific components from multiple domains across multiple treatments, within the context of an overarching theoretical framework, which was derived from the literature.

### Theoretical framework

**Figure 1:**
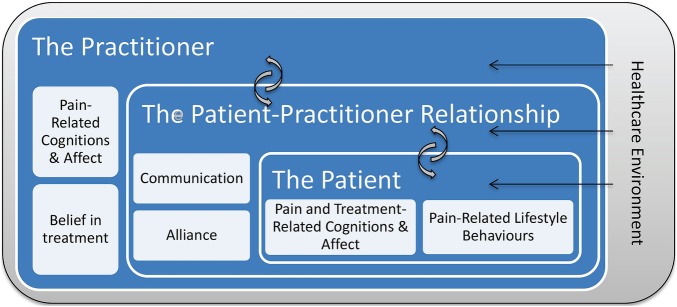
Multilevel framework of non-specific treatment components.

Figure 1 presents the theoretical framework for this study. The patient is at the centre of this model: non-specific components lead to reductions in self-reported pain and disability through their impact on the patients’ pain cognitions/emotions and behaviours. In particular, we hypothesise that non-specific treatment components affect patient outcomes by (1) triggering changes in patients’ cognitive and affective states involved in the maintenance of pain, such as fear avoidance beliefs and/or (2) affecting patients’ self-efficacy for coping with their pain, and/or (3) influencing patients’ health behaviours such as physical activity, diet and coping. These mechanisms may themselves be common across therapies (eg, a direct effect of positive expectations on reported pain outcomes) or may operate via an interaction between non-specific and specific components of treatment (eg, positive expectations lead to patients having a better understanding of the treatment-specific explanations, which then leads to increased uptake of theoretically driven lifestyle advice).

The next layer in the model is the patient–practitioner relationship, two key components of which are communication style and therapeutic alliance. We hypothesise that positive patient–practitioner relationships engender supportive self-care communication and shared goals, which then trigger adaptive changes such as increased self-efficacy for coping and uptake of lifestyle advice. This process has been illustrated qualitatively in acupuncture.^44^ For example, when a practitioner communicates in a patient-centred empathetic way, a patient will feel respected and understood and will experience a supportive bond with their therapist. The patient will be more likely to believe the practitioner's claims that their pain is manageable and to share ownership of the treatment plan, resulting in improved pain beliefs, increased self-efficacy for coping with pain and uptake of lifestyle advice. The interactions between the therapeutic relationship and patients’ beliefs are probably bidirectional: evidence from psychotherapy suggests that the effect of patients’ expectations on outcomes is partially mediated by the therapeutic relationship.^49^ ^50^ In other words, patients who expect therapy to be successful are more open to developing positive patient–practitioner relationships which go on to augment therapy outcomes.

The patient–practitioner relationship is itself partially determined by the individual practitioner, the next layer in the model. Practitioners’ beliefs about the nature of back pain have been shown to influence their clinical decision-making.^24^ ^25^ We hypothesise that practitioners’ beliefs about back pain also influence their communication style. For example, practitioners who have a more biomedical model of back pain are probably less likely to engage in psychosocial talk and hence may be less likely to influence patients’ pain-related affect and cognitions.

Finally, the outer layer of the model comprises the healthcare environment in which the practitioner and patient interact. Environmental factors are hypothesised to affect patients’ outcomes via their impact on the practitioner's behaviour, the therapeutic relationship and the patients themselves. For example, organisational constraints in the NHS encourage managed care, can discourage some practitioners from taking holistic treatment orientations and can foster paternalistic therapeutic relationships, thus reducing patients’ sense of control.^8^ ^9^ ^51^

### Setting

This project uses LBP as a model for studying non-specific treatment components and processes because: LBP is highly prevalent,^52^ there is no gold-standard treatment and existing treatments provide only modest relief,^53^ ^54^ meaning an exploration of non-specific components offers an opportunity to enhance existing treatments. Furthermore, pain theories (eg, fear avoidance model^55^) provide an excellent biopsychosocial framework to inform an understanding of how non-specific components trigger effects. Finally, orthodox and CAM approaches are popular in patients with LBP and are recommended in clinical guidelines,^56–60^ enabling comparisons between orthodox and CAM therapies. We have chosen to focus on physiotherapy (orthodox), osteopathy (CAM) and traditional acupuncture (CAM) as these are commonly used by patients with painful conditions^60–62^ and are recommended in clinical guidelines for the management of LBP.^56^ They have distinct theoretical and explanatory frameworks but all involve some hands-on treatment, a series of treatments over time allowing for the development of a patient–practitioner relationship and an element of patient education regarding self-management.

### Aims

The aims are to:

1. Identify the most powerful non-specific treatment components (ie, those that have the largest effect on patient outcomes).
2. Compare the magnitude of non-specific effects across orthodox (physiotherapy) and CAM (osteopathy, acupuncture) therapies.
3. Test whether theoretically derived mechanistic pathways explain the effects of non-specific components.
4. Identify similarities and differences in patient–practitioner interactions across the three therapies.

The associated hypotheses are presented in table 1.

**Table 1:** Aims and hypotheses

Aim	Associated hypotheses
1. Identify the most powerful non-specific treatment components (ie, those that have the largest effect on patient outcomes)	Patients experience less back-related disability after treatment for LBP when non-specific components are more positive, ie, when: The therapeutic alliance is stronger and practitioner communication is more patient-centred The healthcare environment is experienced by patients as pleasant, accessible and convenient, and by practitioners as supportive Appointment duration is longer Patients expect their treatment to be effective, perceive it as credible and suitable for them personally and have few concerns about it Practitioners have a biopsychosocial orientation to back pain and expect patients to respond well to treatment
2. Compare the magnitude of non-specific effects across orthodox (physiotherapy) and CAM (osteopathy, acupuncture) therapies	CAM therapies (acupuncture and osteopathy) produce larger non-specific effects than orthodox therapy (physiotherapy) Differences between therapies are more pronounced in the NHS than in the private sector^8–10^
3. Test that theoretically derived mechanistic pathways explain the effects of non-specific components	Non-specific components reduce patients’ back-related disability via: Improvements in patients’ pain beliefs (eg, reduced fear of pain) Increases in patients’ self-efficacy for coping with pain Increased implementation of theory-specific lifestyle advice
4. Identify similarities and differences in patient–practitioner interactions across the three therapies	Acupuncture and osteopathy consultations score higher than physiotherapy consultations on an index of ‘patient-centeredness’ Patients who receive consultations that score higher on the patient-centeredness index report more positive outcomes than patients who receive consultations that score lower on the patient-centeredness index Consultations in the private sector score higher than those in the NHS on the patient-centeredness index^12–14^

CAM, complementary and alternative medicine; NHS, National Health Service.

## Methods and analysis

This research comprises a prospective questionnaire-based cohort study with a nested mixed-methods study. The questionnaire-based study addresses aims 1–3. The nested mixed-methods study addresses aim 4. The questionnaire-based study collects self-report data from a large number of patients and practitioners and tests the effects of non-specific components from all five domains. The nested mixed-methods study conducts qualitative and quantitative analyses of a small sample of audio-recorded patient–practitioner interactions, to complement the self-report methods and broader focus of the questionnaire-based study.

### Prospective questionnaire-based cohort study

#### Design

**Figure 2:**
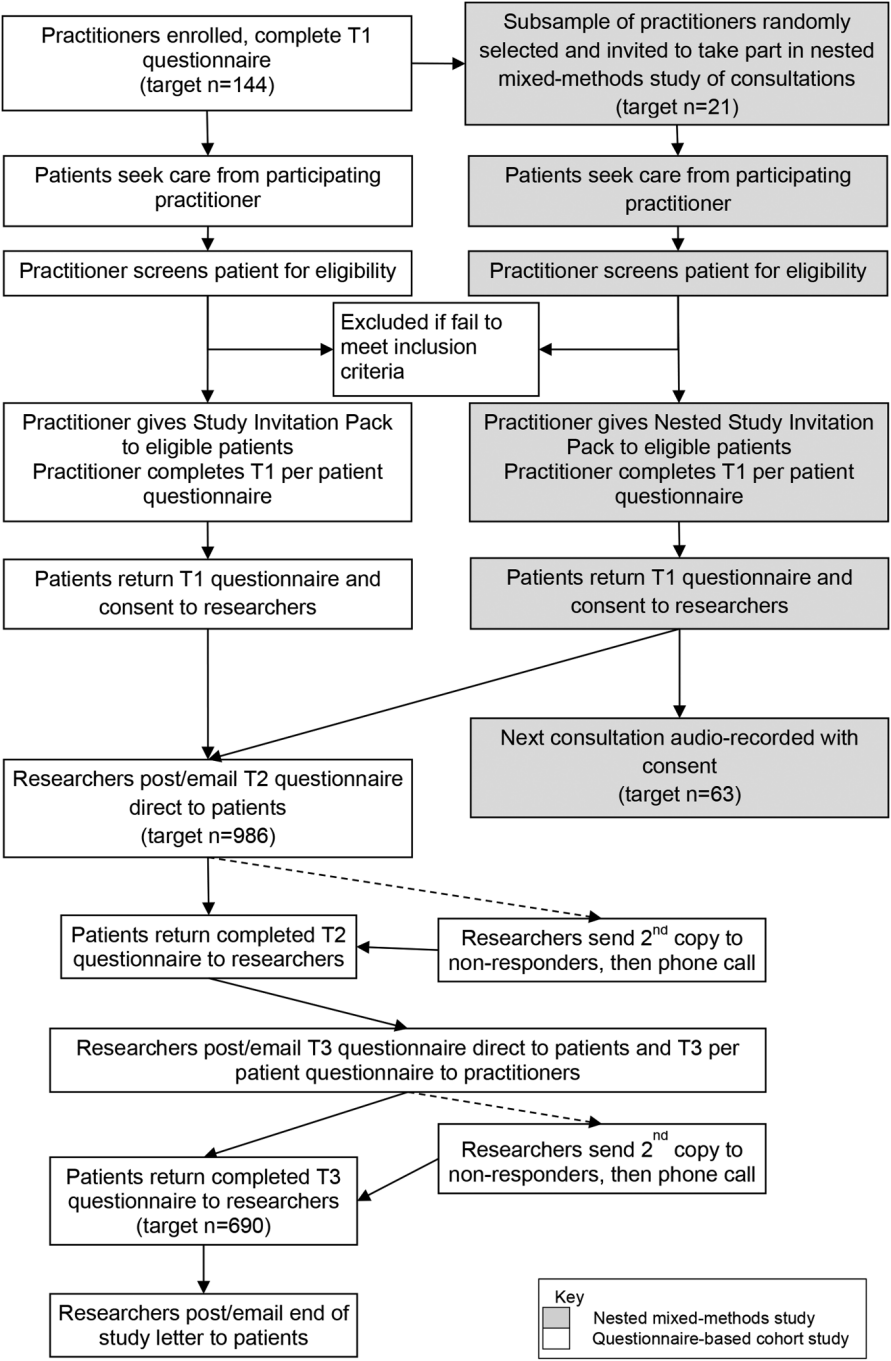
Study flow chart.

Practitioners and patients complete questionnaires at three time points: baseline (T1: after the first consultation for a new episode of LBP), during treatment (T2: 2 weeks post-baseline) and short-term outcome (T3: 3 months post-baseline) (figure 2). Non-specific factors are measured once with time points chosen to reduce ceiling effects, capture data accurately and spread the questionnaire burden. Outcomes, prognostic indicators and potential mediators are measured at T1, T2 and T3, to permit tests of whether scores on non-specific factors are associated with changes over time in prognostic indicators or mediators. Participants can choose to complete hard copies (mailed, returned via Freepost) or electronic copies (emailed, completed online). Online and paper versions of our primary outcome are equivalent, can be used interchangeably and patients value having this choice.^63^

#### Participants

To participate, practitioners must treat at least one patient with LBP on average per week and have at least 3 years relatively current experience in musculoskeletal work. Osteopaths and physiotherapists must be registered with the General Osteopathic Council and Chartered Society of Physiotherapy, respectively. Acupuncturists must be eligible to register with the British Acupuncture Council (BAcC) (ie, a 3-year qualification or equivalent in traditional acupuncture). Physiotherapists who use acupuncture clinically can participate (as physiotherapists) as this is becoming common clinical practice.

To participate, patients must be at least 18 years old, seeking treatment from a participating practitioner at their first consultation for a new episode of LBP and score at least 4 on the Roland-Morris Disability Questionnaire (RMDQ).^64^ Patients will be ineligible if they: are unable to complete questionnaires in English or Welsh, have serious underlying pathology (inflammatory arthritis, malignancy) or have practitioner-identified conditions that would prevent the sought treatment being applied. Arguably, LBP as managed in primary care should be conceptualised as typically having a chronic–episodic timeline with recurrent acute flare-ups against a backdrop of temporary remission or less bothersome symptoms.^65^ ^66^ Broad inclusion criteria thus best reflect the clinical situation and will ensure that our cohort is representative of patients with LBP treated by physiotherapists, osteopaths and acupuncturists.

#### Sample size

Data from our earlier longitudinal questionnaire-based study of acupuncture for LBP were used as the basis for these sample size calculations.^37^ The planned multilevel analysis requires a final sample size of 690 patients to detect correlations between predictors, hypothesised mediators and the primary outcome, the RMDQ,^64^ with Pearson's R=0.2 (and corresponding β regression coefficients of 0.235) with 95% power and p<0.0005 (two tailed). This sample size has been adjusted for clustering of patients within practitioners using a factor of 1+(k−1)×ICC, where k=8 (number of patients per practitioner) and ICC=0.01 (ICC based on UK Beam^67^ ^68^). The final sample size will also be sufficient to detect, within each therapy subgroup, correlations between the predictors and the primary outcome of Pearson's R=0.2, at p<0.04 with a minimum of 81% power. This sample size should also allow us to test for interactions and possible therapy-specific effects.

If the data are not normally distributed and transformations do not achieve a normal distribution, we will still have sufficient power to detect bivariate correlations (eg, Spearman's ρ=0.2) between the predictors and the primary outcome in the whole sample (power=95%, p<0.002) and in each therapy subgroup (power=80%, p<0.05).

If all practitioners recruit 8 patients, then 86 practitioners are needed; however, some attrition is likely. To allow for 40% attrition of practitioners, 144 practitioners will be recruited (48 per therapy). To allow for 30% attrition of patients, 986 patients will be recruited. Practitioners are asked to recruit 10–30 consecutive eligible adult patients, allowing some flexibility and enhancing recruitment to at least the minimum level.

#### Measures

Table 2 summarises the chosen constructs and measures.

**Table 2:** Constructs and measures

Domain	Construct	Measure	Items	Time point*	Completed by
Outcomes					
Primary	Disability	RMDQ^64^	24	T1, T2, T3	Patient
Secondary	Social role disability	Core item^69^	1	T1, T2, T3	Patient
	Work disability	Core item^69^	1	T1, T2, T3	Patient
	Pain	Core item^69^	1	T1, T2, T3	Patient
	Well-being	Core item^69^	1	T1, T2, T3	Patient
	Satisfaction	Core item^69^	1	T1, T2, T3	Patient
Non-specific factors					
Relationship	Therapeutic alliance	WAI-SF^70^ ^71^	12	T2	Patient
Healthcare environment	Organisational	ABS-mp^24^ ^72^	9	T1	Practitioner
	Appointments, access, facilities	PSQ^73^	16	T2	Patient
Treatment characteristics	Modalities	Single item	2	T3 per patient	Practitioner
	Duration	Single item	1	T3 per patient	Practitioner
Patient's beliefs	Treatment beliefs	LBP treatment beliefs questionnaire^74^	16	T1	Patient
Practitioner's beliefs	Attitudes to LBP	ABS-mp^24^ ^72^	12	T1	Practitioner
	Outcome expectations	Single item	1	T1 per patient	Practitioner
Mediators/prognostic indicators					
	Risk complexity for recovery	STarT Back^75^	9	T1, T2, T3	Patient
	Self-efficacy	Self-efficacy for pain management^76^	5	T1, T2, T3	Patient
	Adherence to lifestyle advice	Single item	3	T1, T2, T3	Patient
	Illness perceptions	BIPQ^77^	9	T1, T2, T3	Patient

*T1=baseline (after first treatment for new episode of LBP); T2=during treatment (2 weeks post-baseline); T3=short-term outcome (3 months post-baseline).

ABS-mp, Attitudes to Back Pain Scale—Musculoskeletal Practitioners; BIPQ, Brief Illness Perceptions Questionnaire; LBP, low back pain; PSQ, Patient Satisfaction Questionnaire; RMDQ, Roland-Morris Disability Questionnaire; WAI-SF, Working Alliance Inventory—Short Form.

##### Outcomes

The primary outcome is self-reported back-related disability, measured using the 24-item RMDQ.^64^ Secondary outcomes (pain intensity, well-being, work and social role disability, satisfaction with care) are measured using recommended core single items.^69^

##### Patient–practitioner relationship

The patient–practitioner relationship is operationalised as the therapeutic alliance; this construct is grounded in theory on how patient–practitioner interactions can elicit psychological/behavioural changes,^78^ consistently predicts patient outcomes in psychotherapy^5^ and offers a good fit with CAM. For example, the three dimensions of therapeutic alliance capture egalitarian partnerships, patient participation and individualised care (as collaboration), perceived interpersonal connection and liking (as affective bond) and new holistic insights into illness and treatment (as concordant goals). These aspects of the patient–practitioner relationship have been shown to be important to CAM patients.^8^ ^27^ ^79–83^

Therapeutic alliance is assessed using the client version of the Working Alliance Inventory—Short Form (WAI-SF), which assesses all three dimensions of working alliance with acceptable psychometric properties.^5^ ^70^ ^71^ The patient-rated WAI-SF may have ceiling effects if used after one treatment but tends to remain stable after the second treatment;^5^ it is therefore administered at T2.^78^ ^84^

##### Healthcare environment

The healthcare environment is operationalised as the organisational environment and the sensory–physical environment. Practitioners’ perceptions of the organisational environment are measured using two single items to assess the caseload and waiting list and two subscales from the psychometrically sound Attitudes to Back Pain Scale—Musculoskeletal Practitioners (ABS-mp): putting limits on sessions and perceived connections within the healthcare system.^24^ ^72^

Patients’ perceptions of the organisational and sensory–physical environment are assessed using three subscales of the Patient Satisfaction Questionnaire designed to assess patient perceptions of the quality of primary healthcare in the UK.^73^ The access subscale measures perceptions of interactions with reception staff; the appointment subscale measures the perceived availability of convenient appointments; the facility subscale measures perceptions of the physical environment of the clinic and waiting room.

##### Characteristics of treatment

To assess the general characteristics of treatment, practitioners record for each patient: the treatment given (physiotherapy, osteopathy, acupuncture), individual modalities used (eg, manipulation, mobilisation, needling), the number of appointments attended and their average duration. Patients report how many treatments they have received.

##### Patients’ beliefs

This domain is operationalised as patients’ treatment beliefs, measured using the brief LBP Treatment Beliefs Questionnaire that assesses four dimensions of treatment beliefs: perceived/anticipated effectiveness, credibility, concerns and individualised fit.^74^ It was explicitly designed for use in mixed cohorts of patients with LBP undergoing diverse treatments. This questionnaire is completed at T1 as patients’ expectations regarding effectiveness should be measured early in treatment.^22^

##### Practitioners’ beliefs

This domain is operationalised as practitioners’ outcome expectations and beliefs about LBP. A single-item numerical rating scale measures practitioners’ outcome expectations for each patient. Four subscales from the ABS-mp measure: willingness to engage with psychological issues, confidence and concern over clinical limitations, reactivation of work and activity and belief in an underlying structural cause of pain.^24^ ^72^

##### Prognostic indicators and mediators

The STarT Back screening tool with good predictive validity is used to assess mood, fear, worry and catastrophising.^75^ ^85^ ^86^ The reliable and valid Brief Illness Perceptions Questionnaire (BIPQ) is used to measure eight dimensions of illness perceptions in relation to LBP.^77^ Self-efficacy for coping with LBP is assessed using the five-item Chronic Pain Self-Efficacy for Pain Management subscale.^76^ There is no gold-standard measure of adherence,^87^ and practitioners’ recommendations are likely to be highly personalised to individual patients. Adherence is therefore conceptualised in relation to three broad domains of lifestyle changes (therapeutic exercises, diet and physical activity) and measured using a single item worded to reduce social desirability bias.^88^ Practitioners also report whether they gave theoretically derived lifestyle advice to each patient.

##### Clinical and demographic covariates

Patient-level covariates are as follows: leg pain and shoulder/neck pain bothersomeness (measured using STarT Back^75^), duration of LBP, age, gender, work status, compensation status, comorbidities, co-treatments and socioeconomic status (indicated by postcode). Practitioner-level covariates are as follows: time since qualifying and experience in musculoskeletal care.

#### Procedure

Figure 2 presents the study flow chart, summarising the flow of participants through the study. Physiotherapists, osteopaths and acupuncturists working in the NHS and the private sector throughout the UK are recruited by advertisements (eg, online and in newsletters) and personal invitations, with support from the Clinical Research Network, the Chartered Society of Physiotherapy, the General Osteopathic Council and the BAcC. Practitioners who express an interest in taking part are sent information and consent forms and offered the opportunity to discuss the study with the researchers before providing written informed consent and completing their T1 questionnaires (see table 2). When potentially eligible patients present for treatment, at their first consultation, practitioners hand them a study invitation pack containing an invitation letter, information sheet, consent form and T1 questionnaire. On inviting an eligible patient into the study, practitioners complete the T1 per patient questionnaire with respect to that patient. All completed consent forms and questionnaires are returned directly to the researchers via prepaid envelopes. The researchers email or mail (participant's choice) the T2 and T3 questionnaires to participants who again return them directly to the researchers. If participants do not respond to T2 and/or T3 questionnaires, the questionnaires are resent and participants are contacted by telephone. If no contact is forthcoming at T3, participants are invited to complete the primary outcome measure by telephone.

The following strategies are used to enhance recruitment and retention rates: monthly update emails to practitioners, small gifts (eg, tea bag/pen) and monetary incentives (£5 voucher) for patients, personalised questionnaires, ink-signed cover letters, coloured ink, stamped return envelopes, first class post and follow-up strategy for non-responders.^89–91^

#### Analysis plan

Data obtained from paper questionnaires will be inputted and data entry checked for accuracy (using double entry for 10% of data). All data will be imported into SPSS for preliminary data analysis, which will include checking measurement properties and distributions of questionnaire scores (performing transformations if necessary to achieve normal distributions), examining and dealing with missing data (eg, by multiple imputation) and ensuring the data meet the assumptions of the main analysis (eg, linear relationships between predictors and outcomes). The main analysis will be performed by multilevel methods (eg, Restricted Maximum Likelihood; REML) using appropriate statistical software (eg, MLWin) to construct a multilevel regression model taking into account the clustering of individual patients within practitioners (two-level model: level 1=patients and level 2=practitioners). As a secondary aim, the self-reported patient outcomes can be modelled as time-varying repeated measures, while the non-specific factors remain time-invariant predictors (three-level model: level 1=time, level 2=patients and level 3=practitioners). We will test for main effects of the predictors (hypothesis 1), interaction effects (hypothesis 2) and mediation effects (hypothesis 3). Multilevel modelling provides an ideal framework for examining such a complex data set and testing our hypotheses, which involve main effects as well as complex interactions between the variables.

### Nested mixed-methods study of consultations

#### Design

This nested study explores whether CAM and orthodox therapists use different verbal communication styles and the extent to which these are more or less effective. A mixed-methods design combines qualitative and quantitative analyses of audio-recorded consultations from a random sample of participants in the questionnaire-based study using each therapy. We have selected audio-recording as the least intrusive and most cost-effective method of observing a consultation, but acknowledge that this method is limited to capturing verbal communication only. The analysis will test specific hypotheses (see table 1), and identify similarities and differences between acupuncture, osteopathy and physiotherapy consultations on (1) the frequency of different types of communication and (2) the thematic content of the consultations.

#### Participants

Using random number tables, we will invite a random sample of practitioners from the questionnaire-based study to take part. Given qualitative evidence suggesting that consultations in the private sector might be more patient-centred than those in the NHS,^8–10^ we will stratify for the healthcare sector.

#### Sample size

We will audio-record 21 consultations from each therapy: seven therapists per therapy will record a single consultation from each of three patients. This should ensure that particularly unusual cases do not dominate and is sufficient to detect a large difference between the therapies with 80% power and α=0.05. (We could locate no previous studies comparing observed CAM and orthodox consultations to inform a more precise power calculation).

#### Procedure

Using random number tables, a random selection of practitioners from the questionnaire-based study will be sent information about this nested study and invited to take part (additional informed consent is taken specifically for this nested study). Consecutive eligible patients who consult practitioners in this nested study will be given study invitation packs including all standard documents for the questionnaire-based study and an additional information sheet and consent form regarding this nested study, requesting written informed consent to audio-record a consultation. On receiving patient consent, the researchers notify the practitioner who audio-records the next consultation using a digital audio-recorder. Patients and practitioners then continue in the Prospective Questionnaire-Based Cohort Study as described above (see Figure 2).

#### Analysis plan

Audio-recordings will be coded with the widely used Roter Interactional Analysis System (RIAS),^92^ which has previously been used for back pain consultations.^93^ ^94^ The RIAS can be applied directly to video or audio-recordings of consultations and requires the coder to rate each expressed meaning with a single code. Codes are mutually exclusive and comprehensive and incorporate task-focused and socioemotional elements of patient–practitioner interactions. We will generate frequency counts for different categories and subcategories of utterances (eg, emotional talk, empathy and concern).^95^ We will combine frequency counts and calculate a ratio of patient-centred to doctor-centred talk,^96^ to produce a patient-centred index for each consultation. A proportion of RIAS coding will be done independently by two coders to check reliability.

A 3×2 analysis of variance (ANOVA) will test for the effects of therapy (osteopathy, physiotherapy, acupuncture) and healthcare sector (NHS, private) on patient-centredness. Regression analyses will test whether patient-centred communication predicts patient outcomes (derived from the questionnaire-based study). Inductive qualitative analysis will explore the thematic content of talk^97^ and take a more holistic view of the consultations, thus addressing some of the limitations of relying solely on quantitative interactional analysis systems^98–100^ and helping capture any unique features of CAM consultations.

## Ethics and dissemination

### Ethics and governance

The study is conducted in accordance with the British Psychological Society Code of Human Research Ethics, the Helsinki Declaration, the Research Governance Framework for Health and Social Care, the Data Protection Act 1998 and International Conference for Harmonisation of Good Clinical Practice (ICH GCP) guidelines. Site-specific approvals are obtained for all NHS sites as required. The study sponsor is the University of Southampton.

Potential benefits of taking part include the chance to reflect on thoughts about treating LBP (practitioners) and the chance to reflect on thoughts about LBP, treatment and health in general (patients). The main disadvantage is the time required to complete the questionnaires. The content of the questionnaires is unlikely to be distressing.

Practitioners from across the UK and their patients are invited to take part on a voluntary basis. Recruitment material makes no therapeutic promises and there is no coercion. Potential participants receive written information that describes the study in detail including: purpose, study procedures, potential risks and benefits to the participant, funding and review arrangements, confidentiality, dissemination plans, what to do if there is a problem and contact details for further information. They have the opportunity to discuss the study and provide full written informed consent before being enrolled. They have the right to withdraw from the study at any time without giving a reason and without penalty.

All data are collected and retained in accordance with the Data Protection Act 1998. Electronic data are stored on University of Southampton secure research filestore. Digital audio-recordings of consultations are password-protected and stored securely electronically for the duration of the research; personal details are removed during transcription. Direct quotes from the audio-recordings will be anonymised before being published (with consent—participants are asked for consent to being quoted verbatim in published reports). Anonymised data will be stored securely for a period of 10 years from study publication, in line with the host institution's guidance. Audio-recordings and personal details necessary to administer the research will be destroyed on study publication.

Small non-monetary (eg, tea bag/pen) and monetary incentives (worth £5) are used in this study. They are non-contingent on response and are presented as small gifts of thanks to participants. The small absolute value of these incentives is very unlikely to exert pressure on potential participants to take part.

### Patient public involvement

Three patient volunteers with experience of musculoskeletal pain and/or at least one of the treatments under study provided feedback in the early stages of developing this project. We have recruited one patient volunteer to contribute to the study on an ongoing basis. Their remit is to comment on study design and implementation issues in order to ensure that our project is sensitive to patients’ concerns and priorities; in particular, to comment on all materials to be given to patients in the studies; to assist in interpreting qualitative themes and quantitative results; to advise on and potentially contribute to dissemination activities.

### Dissemination

Results will be disseminated at conferences and in peer-reviewed journals. Regardless of the results, the questionnaire study and the nested mixed-methods study will be published. We will also disseminate findings to all participants, to relevant professional bodies and patient organisations and to the general public. We will provide personalised feedback to the practitioners in the nested study of the consultations based on the RIAS analysis of their communication. We will hold a stakeholder workshop to discuss the implications of our findings for patients, practice and policy.

## Discussion

This research is examining the role of non-specific treatment components in orthodox and CAM management of LBP. In doing so, we will come to better understand the nature and effects of non-specific components. This is a vital next step to enable research on non-specific treatment components to contribute to enhancing treatment to help people remain active and pain-free. Our research will identify the most effective non-specific treatment components in LBP and model how they produce positive patient outcomes; this will help practitioners, policymakers and researchers to optimise non-specific components across diverse therapies, thus enhancing treatments and maximising patient benefit. Depending on the results, we hope to be able to suggest how non-specific components of orthodox treatments will be enhanced by learning from CAM.
